# Preferential utilization and colonization of keratin baits by different myco-keratinophiles

**DOI:** 10.1186/s40064-016-2874-1

**Published:** 2016-07-28

**Authors:** Sandeep Kotwal, Geeta Sumbali

**Affiliations:** Department of Botany, University of Jammu, Jammu, 180 006 India

**Keywords:** Feathers, Keratinophilic fungi, Keratin baits, Preferential utilization

## Abstract

Myco-keratinophilic species have a predilection for different keratinous substrates but show variability in their affinity towards them. Keeping this in view, a survey was conducted in the Khardung and Khardung La soils of Ladakh (India) and 28 myco-keratinophilic species belonging to 15 fungal genera (*Sarocladium*, *Aspergillus*, *Beauveria*, *Chrysosporium*, *Cladosporium*, *Alternaria*, *Epicoccum*, *Fusarium*, *Gibberella*, *Clonostachys*, *Paecilomyces*, *Purpureocillium*, *Metarhizium*, *PenicilliumandSagenomella*) were isolated by using keratin bait technique. These isolated species were tested for their preferential utilization ability and colonization on different baits by morphological assessment. Different types of keratin baits used were feathers, human hair, human nails and wool. Overall assessment revealed that feathers were colonized and utilized by all the species (100 %), followed in decreasing order by nails (89.29 %), hair (85.71 %) and sheep wool (67.86 %). So, it is concluded that feather baiting technique, could be more useful in trapping keratinophilic fungi than the hair baiting technique which is till date regarded as the best method for the isolation of myco-keratinophiles. On the basis of succession on keratinous baits, the recovered keratinophilic species were also categorized into four categories: early successional species (pioneer colonizers), late successional species (final colonizers), persistent species and no-pattern species.

## Background

Keratinophilic fungi are considered as an ecologically important group of highly specialized fungi, which are adapted to the utilization of keratin as the main or sole source of nutrition (Kunert 2000). This unique fungal group is attracting lot of attention throughout the world because of their ability to degrade hard keratin, which is otherwise resistant to degradation by most of the other microorganisms (Filipello 2000). In nature, they exist as self sufficient saprophytes as long as environmental conditions are favourable but they may become parasitic by accident and then pathogenic. Soils rich in keratinous material are found to be most conducive for the growth and occurrence of keratinophilic fungi (Otcenasek 1978; Mercantini et al. 1980). But occurrence of keratinophilic fungi also depends upon various genomic and climatic factors such as organic matter, soil humidity, pH, temperature, soil texture, depth of soil profile and other microorganisms (Srivastava et al. 1990). For isolation of keratinophilic fungi from soil, keratin or hair baiting technique given by Vanbreuseghem (1952) is widely used. As per this procedure, different keratinous substrates are used as a bait to lure keratinophilic species. However, different species vary in their preference for colonization and utilization of these baits. Keeping this in view, an experiment was carried out to find out the best keratin baits for trapping these fungi and to know the successional pattern of the isolated species.

## Methods

Keratin rich substrates found abundantly in nature were used as baits. These included feathers, human hair, human nails and sheep wool. Collected baits were thoroughly washed with water, air dried at room temperature and then cut into small pieces. Finally, they were washed with 70 % alcohol, air dried and sterilized by autoclaving at 15 lbs./sq inch for 20 min. Petridishes were half-filled with soil and sterilized in an oven at 180 °C for 4–5 h on three successive days. Thereafter, 7 days old culture of the test fungus growing in a test tube was scrapped in 5 ml sterile water and added to the sterile soil contained in the Petridish inoculated with test culture. These were incubated at 28 ± 2 °C for 20 days and at the end of this period, degree of colonization and preferential utilization of the baits by keratinophilic fungal species was recorded. Morphological appearance of colonized and invaded keratin baits was examined under light microscopy using direct samples.

## Results and discussion

After incubation period, the keratin baits were visually examined for the growth of keratinophilic fungal species and the results are presented in Table 1. Perusal of data shows that keratinophiles differ in their substrate preferences for colonization. Out of the four different keratinous baits used, feathers were colonized by all the recovered keratinophilic species (28), human nails by 25 fungal species, human hair by 24 fungal species and sheep wool by 19 fungal species (Table 1). Earlier, Sundaram (1987) also reported sheep wool as a poor bait in comparison to bird feathers and human hair.

**Table 1:** Preferential colonization of keratin baits by mycokeratinophiles

Myco-keratinophilic species	Keratin baits used			
	Feathers	Human hair	Human nails	Sheep wool
*Sarocladium* *bacillisporum*	++	+	++	+
*S*. *implicatum*	+++	+++	+++	++
*Aspergillus* *flavus*	+++	++	+++	+
*A*. *parasiticus*	+++	++	++	+
*A*. *sydowii*	++	++	+++	−
*A*. *ustus*	++	++	+++	+
*A*. *wentii*	+	+	−	−
*Beauveria* *bassiana*	+++	++	+++	+
*Chrysosporium* *inops*	++	+	++	+
*C*. *merdarium*	+++	+++	+++	++
*C*. *queenslandicum*	+++	+++	+++	++
*Chrysosporium* *anamorph* *of* *Gymnoascus* *demonbreunii*	++	+	++	+
*Cladosporium* *cladosporioides*	++	+	++	+
*Alternaria* *chlamydosporigena*	++	−	++	+
*Aspergillus* *stellatus*	++	+	−	−
*Epicoccum* *nigrum*	+++	++	+	−
*Fusarium* *oxysporum*	+++	+++	++	+
*F*. *sporotrichioides*	+++	++	+++	+
*F*. *trichothecioides*	++	−	+	−
*F*. *incarnatum*	++	−	++	−
*Gibberella* *fujikuroi*	++	+	++	+
*Clonostachys* *rosea*	++	++	++	+
*Purpureocillium* *lilacinum*	+++	+++	++	+
*Metarhizium* *marquandii*	+++	++	+++	+
*Paecilomyces* *divaricatus*	+++	++	++	+
*Penicillium* *brevicompactum*	+++	++	+	−
*P*. *griseofulvum*	++	+	−	−
*Sagenomella* *alba*	++	−	+	−
Total number of fungal species colonizing individual baits	28	24	25	19

+++, excellent growth; ++, medium growth; +, slow growth; −, no growth

**Fig. 1:**
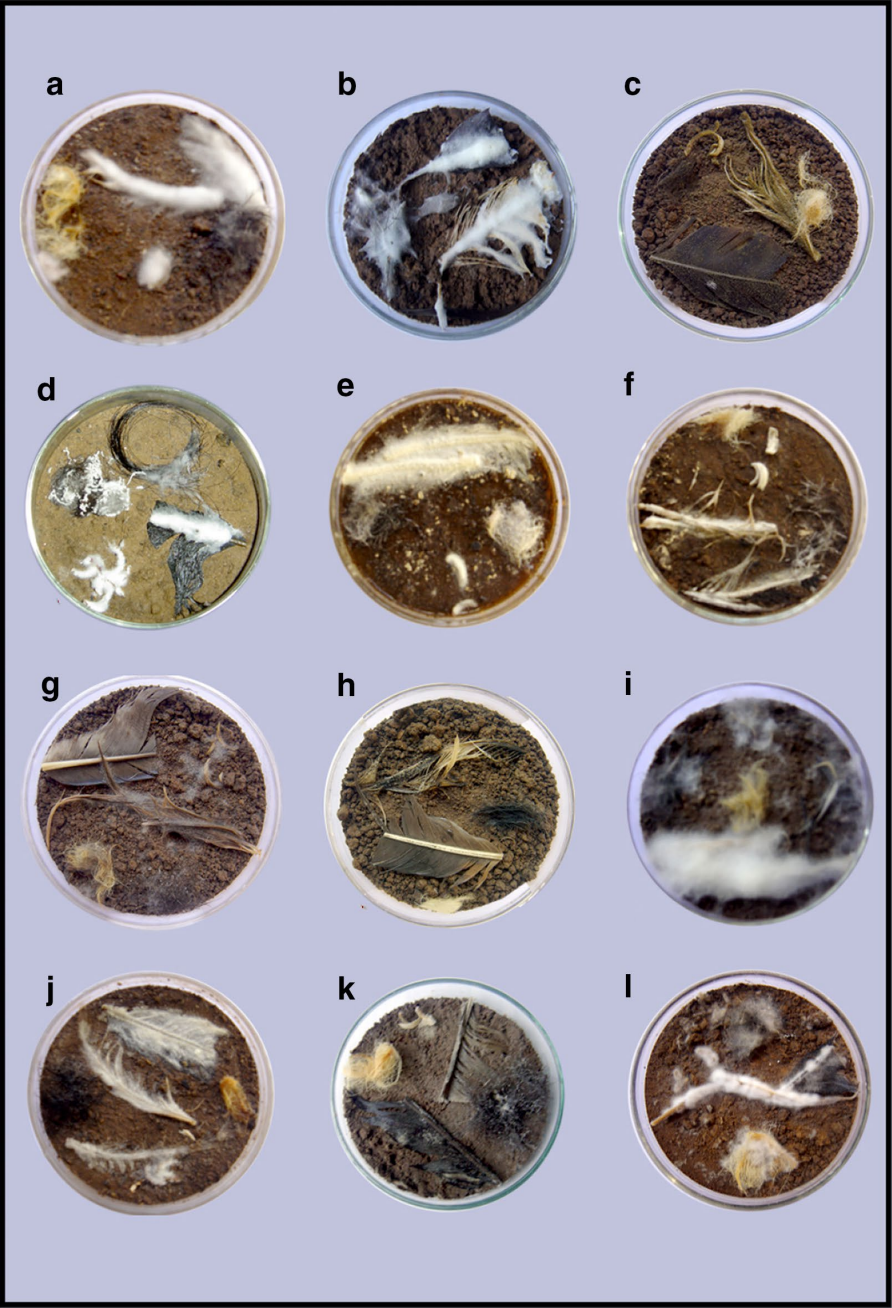
Differential colonizing ability of **a** *Sarocladium* *bacillisporum*, **b** *Sarocladium* *implicatum*, **c** *Aspergillus* *parasiticus*, **d** *Beauveria* *bassiana*, **e** *Chrysosporium* *merdarium*, **f**. *Chrysosporium* *queenslandicum*, **g** *Alternaria* *chlamydosporigena*, **h** *Epicoccum* *nigrum*, **i** *Fusarium* *oxysporum*, **j** *Fusarium* *sporotrichioides*, **k** *Clonostachys* *rosea*, **l** *Metarhizium* *marquandii*

In the present investigation, substrate specificity was found to be variable within the species of the same genus also. Among the recovered *Chrysosporium* species, *C*. *merdarium* and *C*. *queenslandicum* showed luxuriant growth and maximum degradation of all the keratinous baits (Fig. 1), whereas *Chrysosporium* anamorph of *Gymnoascusdemonbreunii* and *C*. *inops* showed moderate growth on bird feathers and human nails but slow growth on human hair and sheep wool. The ability of *Chrysosporium* species to colonize all the keratinous substrates confirmed the cosmopolitan nature of this genus. Since, all the *Chrysosporium* species were observed to be growing either luxuriantly, moderately or slowly on various keratinous residues, it is probable that they possess specific enzymatic system for degradation of keratin and it is, therefore, important to recognize their potential as opportunistic pathogens. Moreover, these fungi by virtue of their ability to colonize and degrade various keratinous substrates are able to add carbon, nitrogen and sulphur content to the soil.

Among *Sarocladium* species, *S*. *implicatum* showed excellent growth on feathers, human hair and human nails but medium growth on sheep wool (Table 1), whereas *S*. *bacillisporum* showed medium growth on feathers and human nails but slow growth on human hair and sheep wool (Fig. 1).

**Fig. 2:**
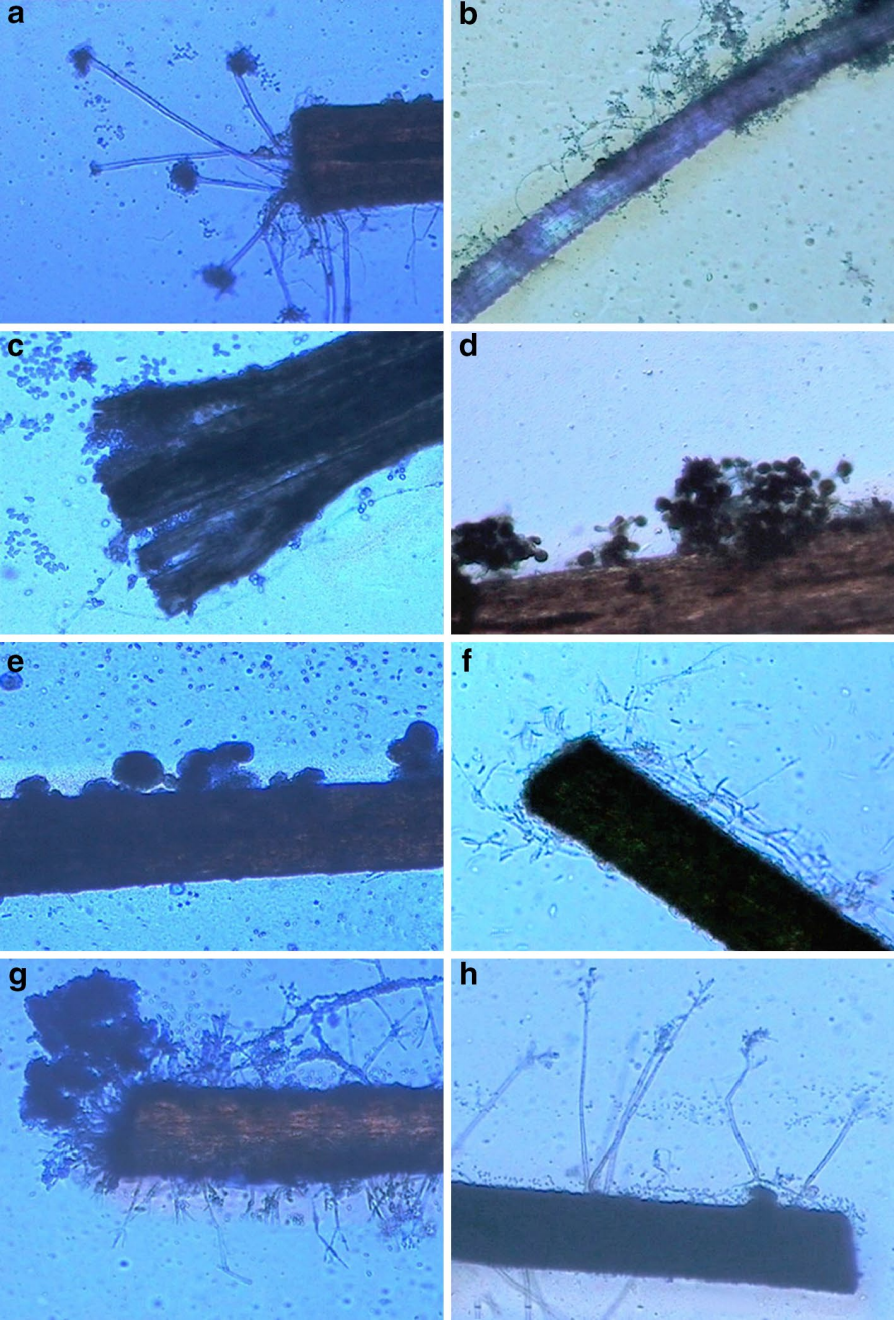
showing colonization of various keratinous baits by some keratinophilic fungal species. **a** Conidiophores of *Aspergillus* *ustus* arising from a hair shaft. **b** Sporogenous cells of *Beauveria* *bassiana* arising from the colonized sheep wool. **c** Degradation of feather by the growth of *Chrysosporium* *merdarium*. **d** Conidiophores and conidia of *Epicoccum* *nigrum* arising from the slightly damaged hair shaft. **e** Hair shaft colonized by cleistothecia of *Aspergillus* *stellatus*. **f** Mycelium and phialides of *Fusarium* *sporotrichioides* arising from the colonized hair shaft. **g** Thick growth of conidiophores of *Clonostachys* *rosea* arising from the slightly damaged hair shaft. **h** Typical conidiophores of *Purpureocillium* *lilacinum* arising from the colonized hair shaft

Different species of *Aspergillus* also depicted keratin specificity. Among the recovered species of *Aspergillus*, *A*. *flavus* and *A*. *parasiticus* showed excellent growth on feathers and human nails but showed moderate growth on human hair and slow growth on sheep wool (Fig. 1). On the other hand, *A*. *sydowii* and *A*. *ustus* grew luxuriantly on human nails and showed moderate growth on feathers and human hair (Fig. 2). However, *A*. *ustus* showed less growth on sheep wool, whereas *A*. *sydowii* showed no preference for this bait. *A*. *wentii* and *A*. *stellatus*, which showed low frequency in Khardung soils could not grow on nails and wool but were slow colonizers of feathers and hair.

Among *Fusarium* species, *F*. *oxysporum* and *F*. *sporotrichioides* were excellent colonizers of feathers, nails and hair but were poor colonizers of sheep wool (Fig. 1), whereas two, other species, *F*. *incarnatum* and *F*. *trichothecioides* showed moderate growth on feathers and nails and no preference for human hair and sheep wool. *Gibberellafujikuroi* showed moderate to low growth on all the investigated keratin baits.

*Purpureocilliumlilacinum*, *Metarhiziummarquandii* and *Paecilomycesdivaricatus*, also showed more preference for feathers than for human nails and hair and were poor colonizers of sheep wool (Table 1). Similarly, *Penicillium* species also showed differential colonizing ability on varied baits. *P*. *brevicompactum* could grow luxuriantly on feathers, moderately on human hair, sparsely on human nails but failed to grow on sheep wool. *P*. *griseofulvum* grew moderately on feathers, sparsely on human hair but could not utilize human nails and sheep wool (Table 1). *Clonostachysrosea* usually showed moderate preference for most of the keratinous baits that were tested (Fig. 1).

Other isolated keratinophilic species, viz., *Beauveriabassiana*, *Cladosporiumcladosporioides*, *Alternariachlamydosporigena*, *Epicoccumnigrum* and *Sagenomellaalba* also showed luxuriant growth on feathers and its maximum degradation (Figs. 1, 2). Among these, *Alternariachlamydosporigena* and *Sagenomellaalba* were not able to grow on human hair, whereas *Epicoccumnigrum* and *Sagenomellaalba* were not able to colonize sheep wool.

The ability of most of the recovered keratinophilic fungi to grow and hydrolyze feather keratin more efficiently than other baits suggests their use in isolating this group of fungi from the soil rather than using the earlier hair baiting technique of Vanbreuseghem (1952). Earlier Pugh (1971), Jain and Agrawal (1980) and Kaul and Sumbali (1994) also observed preferential utilization and maximum degradation of feathers by most of the isolated keratinophiles. Further, moderate to low growth of some of the recovered myco-keratinophiles on different baits suggests that they have low keratinase producing ability or it may be due to the biochemical differences existing in the keratin of various ectodermal appendages. Similar observations have been recorded by Kunert (2000), who found that in case of hard keratin (α-keratin), the rate of hydrolysis corresponds roughly to hardness, that is, cystine content and disulphide bonds. Therefore, since feathers and nails have lesser cystine content and thus few disulphide bonds, they are more easily cleaved by keratinophilic fungi than human hair and sheep wool.

Excellent colonization of all the keratinous substrates was shown mostly by *Chrysosporium* species and *Sarocladiumimplicatum*, indicating that they possess specific enzymatic system for degradation of keratin. Therefore, it is important to recognize their potential as pathogens. Substrate specificity was also found to be variable within the species of the same genus.

On the basis of their succession on keratinous baits the, keratinophilic mycobiota were categorized into four categories.

1. Early successional species (pioneer colonizers):
  Species which appear only at the beginning (within 15 days after soil was baited with keratin-baits) included *Sarocladiumimplicatum*, *Aspergillusparasiticus*, *A*. *sydowii*, *A*. *ustus*, *A*. *stellatus*, *Beauveriabassiana*, *Fusariumoxysporum*, Clonostachys rosea and *Penicilliumgriseofulvum*.
2. Late successional species (final colonizers):
  Species which appear after 15 days of the incubation period included *Chrysosporiummerdarium*, *C*. *queenslandicum*, *C*. *inops* and *Chrysosporium* anamorph of *Gymnoascusdemonbreunii*.
3. Persistent species:
  Species which are present persistently on the keratinous baits e.g., *Aspergillusflavus*, *Fusariumsporotrichioides*, *Purpureocilliumlilacinum*, *Metarhiziummarquandii*, *Paecilomycesdivaricatus*, and *Penicilliumbrevicompactum*.
4. No-pattern species:
  Species that did not seem to have a clear successional pattern. These included *Sarocladiumbacillisporum*, *Aspergilluswentii*, *Cladosporiumcladosporioides*, *Alternariachlamydosporigena*, *Epicoccumnigrum*, *Fusariumtrichothecioides*, *F*. *incarnatum* and *Sagenomellaalba*.

Similar results were obtained by Ali-Shtayeh and Jamous (2000) while working on the succession of human hair by keratinophilic mycobiota of the soil. As observed during this investigation, Ali-Shtayeh and Jamous (2000) also found *Beauveriabassiana* and *Penicilliumgriseofulvum* as the early pioneer colonizers. In addition, as noted in the present investigation, even De Vries (1952) observed that the final fungal colonizers of keratinous baits were the typical keratinophilic hyphomycetes such as species of *Chrysosporium*.

Overall assessment of the recovered myco-keratinophiles for their growth on four different keratinous baits revealed that feathers were colonized and utilized by all the species (100 %), followed in decreasing order by nails (89.29 %), hair (85.71 %) and sheep wool (67.86 %). Similar observations have been recorded by Kunert (2000) who found that the rate and completeness of the degradation is dependent on the kind of substrate and correspond roughly to its hardness, that is, cystine content. Therefore, human, dog, horse and cattle hairs are attacked more slowly than the feathers of birds.

## Conclusions

In view of these observations, it is concluded that hair baiting technique as given by Vanbreuseghem (1952) and recorded by many other workers (Sundaram 1987; Gugnani et al. 2012; Pakshir et al. 2013; Sarkar et al. 2014; Soleymani et al. 2015; Sharma and Choudhary 2015**)** as the best method for the isolation of myco-keratinophiles is not very true. Instead, as observed during the present investigation, feather baits, which could allow the growth of all the recovered keratinophiles are more useful in trapping this unique group.

It was also recorded that the isolated myco-keratinophiles showed a second preference for nails, which indicates that they possess an ability of efficiently hydrolyzing the nail keratin. Therefore, they may pose a potential threat for onychomycosis, particularly among the farmers, gardeners, children and old people.
